# Machine Learning in Multimodal Medical Imaging

**DOI:** 10.1155/2017/1278329

**Published:** 2017-03-05

**Authors:** Yong Xia, Zexuan Ji, Andrey Krylov, Hang Chang, Weidong Cai

**Affiliations:** ^1^Shaanxi Key Lab of Speech & Image Information Processing (SAIIP), School of Computer Science, Northwestern Polytechnical University, Xi'an 710072, China; ^2^School of Computer Science and Engineering, Nanjing University of Science and Technology, Nanjing 210094, China; ^3^Faculty of Computational Mathematics & Cybernetics, Lomonosov Moscow State University, Moscow, Russia; ^4^Berkeley Biomedical Data Science Center (BBDS), Biological Systems and Engineering Division, Lawrence Berkeley National Laboratory, Berkeley, CA 94720, USA; ^5^Biomedical and Multimedia Information Technology (BMIT) Research Group, School of Information Technologies, University of Sydney, Sydney, NSW 2006, Australia

Machine learning techniques have been increasingly applied in the medical imaging field for developing computer-aided diagnosis and prognosis models. Multimodal medical imaging can provide us with separate yet complementary structure and function information of a patient study and hence has transformed the way we study living bodies. Therefore, using machine learning techniques to deal with multimodal medical images is much more challenging due to the diversity of biophysical-biochemical mechanisms. In these years, researchers mainly adapt modern machine learning and pattern recognition techniques such as supervised, unsupervised, semisupervised, and deep learning to solve multimodal medical imaging related problems.

To record the ideas of talents and gather more contributions to these fields, this special issue was launched and supported by this journal. This special issue focuses on the new imaging modalities/methodologies and new machine learning algorithms/applications for the further development in the multimodal medical imaging field, which will provide opportunities for academics and industrial professionals to discuss the latest issues and progresses in the area of multimodal medical imaging. The papers contained in this special issue address the development and application of medical image segmentation, registration, fusion, classification, image restoration, image retrieval, and computer-aided diagnosis.

In “Estimation of Response Functions Based on Variational Bayes Algorithm in Dynamic Images Sequences,” B. Shan proposes a nonparametric Bayesian model to estimate the response functions in dynamic medical imaging, in which the nonparametric Bayesian priors are designed to favor desirable properties of the functions and used to improve the estimation of response functions.

In “Two-Layer Tight Frame Sparsifying Model for Compressed Sensing Magnetic Resonance Imaging,” S. Wang et al. propose a two-layer tight frame sparsifying model for compressed sensing magnetic resonance imaging (MRI) by sparsifying the image with a product of a fixed tight frame and an adaptively learned tight frame, which is solved by a three-level Bregman numerical algorithm and enables accurate MRI reconstruction from highly undersampled data with efficiency.

In “Many is Better than One: An Integration of Multiple Simple Strategies for Accurate Lung Segmentation in CT Images,” Z. Shi et al. present a novel computerized tomography (CT) lung image segmentation method by integrating multiple strategies, including the guided filter to smooth the image, the optimized threshold to get binary image, region-growing strategy to extract thorax regions, and random walk algorithm to segment lung regions and to get the state-of-the-art segmentation accuracy.

In “Pulmonary Nodule Detection Model Based on SVM and CT Image Feature-Level Fusion with Rough Sets,” T. Zhou et al. present a pulmonary nodules detection algorithm based on support vector machine (SVM) and CT image feature-level fusion with rough sets to improve the detection accuracy of pulmonary nodules in CT image. Both the unreasonable feature structure and the nontightness of feature representation are taken into consideration in this pulmonary nodules detection algorithm.

In “Multigrid Nonlocal Gaussian Mixture Model for Segmentation of Brain Tissues in Magnetic Resonance Images,” Y. Chen et al. propose a novel segmentation method based on the regional and nonlocal information to overcome the impact of image intensity inhomogeneities and noise in human brain magnetic resonance images.

In “DTI Image Registration under Probabilistic Fiber Bundles Tractography Learning,” Z. Guo et al. propose a diffusion tensor imaging (DTI) image registration method under probabilistic fiber bundles tractography learning, where the residual error model is modified with finite sample set and the calculated deformation field is then registered on the DTI images.

In “Automated Segmentation of Coronary Arteries based on Statistical Region Growing and Heuristic Decision Method,” Y. Tian et al. propose a fully automated coronary artery segmentation from cardiac data volume based on a statistics region growing together with a heuristic decision to further help cardiovascular radiologists detect and quantify stenosis.

In “Rapid Retrieval of Lung Nodule CT Images Based on Hashing and Pruning Methods,” L. Pan et al. propose a new retrieval framework based on a hashing method for lung nodule CT images, which can translate high-dimensional image features into a compact hash code to greatly reduce the retrieval time and memory space. Moreover, a pruning-based decision rule is utilized in this algorithm to improve its retrieval precision.

In “The Classification of Tongue Colors with Standardized Acquisition and ICC Profile Correction in Traditional Chinese Medicine,” Z. Qi et al. design a tongue color classification approach using a standardized tongue image acquisition process, color correction, and several machine learning techniques for tongue inspection-based diagnosis in traditional Chinese medicine.

In “Diagnostic Method of Diabetes Based on Support Vector Machine and Tongue Images,” J. Zhang et al. develop a SVM-based diagnostic method for diabetes using standardized tongue images. This work shows the potential of applying digitalized tongue images, which are usually used in traditional Chinese medicine, to the diagnosis of diabetes.

In “A Computer-Aided Analysis Method of SPECT Brain Images for Quantitative Treatment Monitoring: Performance Evaluations and Clinical Applications,” X. Zheng et al. introduce and validate a computer-aided analysis method to achieve the quantitative treatment monitoring based on single-photon emission computed tomography (SPECT) images, which can provide a convenient solution to generate a parametric image and derive the quantitative indexes from the longitudinal SPECT brain images for treatment monitoring.

The papers in this special issue provide a useful message of machine learning techniques in dealing with multimodal medical images. This unique and informative collection of papers highlights the direction of related studies. This special issue illustrates the important role that machine learning techniques play in the multimodal medical imaging fields.

